# Carrageenan as a dry strength additive for papermaking

**DOI:** 10.1371/journal.pone.0171326

**Published:** 2017-02-07

**Authors:** Zhenhua Liu, Xinping Li, Wei Xie

**Affiliations:** 1 School of Chemical Engineering, Wuzhou University, Wuzhou, Guangxi Province P. R. China; 2 College of Bioresources Chemical and Materials Engineering, Shanxi University of Science & Technology, Xi’an, Shanxi Province P. R. China; College of Agricultural Sciences, UNITED STATES

## Abstract

Carrageenans are commercially important sulfated gums found in various species of red seaweeds (Rhodophyta), wherein they serve a structural function similar to that of pectins in land plants. In this study, carrageenan was used independently or in combination with cationic polyacrylamide (CPAM) and/or Al_2_(SO_4_)_3_ to explore its application as a dry strength additive in papermaking. Strength index determination, ash content detection, FTIR characterization and SEM observation were performed on prepared handsheets. The results showed that with 0.6% Al_2_(SO_4_)_3_ and 0.2% carrageenan as additives, the tensile index increased by 13.53% and precipitated calcium carbonate (PCC) retention increased by 57.06%. With 0.6% Al_2_(SO_4_)_3_, 0.2% carrageenan and 0.03% CPAM as additives, PCC retention increased by 121% while the tensile index did not fall compared to handsheets without additives, indicating that carrageenan could enhance the strength of handsheets and be used as an anionic dry strength agent.

## Introduction

Dry strength additives are important chemicals in the papermaking industry to increase paper strength. Many water-soluble polymers, which can form hydrogen bonds with cellulose fibres, can be used as dry strength additives. Dry strength additives are typically used to offset the decline of paper strength resulting from addition of fillers or secondary fibres (such as recycled fibres). Frequently used dry strength additives are natural or synthetic polymers. Natural polymers include starch (cationic/anionic starch) and gums (guar gum, for example). Synthetic polymers comprise polyacrylamide and its derivatives, polyvinyl alcohol, etc. In most cases, a mass fraction of 0.1%-0.35% of these substances can achieve an effective dry strengthening effect. Thus far, most studies focused on the use of synthetic polymers, starch or terrestrial plant gums in papermaking [1–5]. As a gum from marine plants, carrageenan used in papermaking has been reported rarely.

Carrageenan is a natural, sulfated polysaccharide extracted from red algae. In addition to mucilaginous components such as carrageenan, other polysaccharides of red algae, such as cellulose, have been previously employed to make paper or nanomaterials [6, 7]. Carrageenan has traditionally been used in various commercial applications such as cosmetics, medicine, and especially in food products as a gelling, thickening, or stabilizing agent [8–11]. The average relative molecular mass of carrageenan is above 100 kD. The linear backbone of carrageenan is formed by alternating units of galactose and 3,6-anhydrogalactose. The linkages in and between the alternating units are β-1,4 and α-1,3 glycosidic bonds, respectively. Approximately 15 to 40% of the monosaccharides in carrageenan are sulphated, which define the anionic feature of carrageenan. According to the position and number of sulfate groups, carrageenan is classified into several types such as λ, κ, ι, ε, and μ [12]. Carrageenan is a widely abundant, environmentally friendly and sustainable material. Although its price is now sometimes higher than other papermaking aid like guar gum, carrageenan is extracted from marine algaes, which doesn’t compete for land for planting crops, As a natural high molecular polymer, carrageenan is presumed to function in improving paper mechanical strength similar to guar gum and may alleviate a growing land demand by bombing population.

## Materials and methods

### Materials

Carrageenan (κ type) was purchased from China Fujian Greenfresh Food Co., Ltd.. Guar gum was obtained from Shree Ram Gum Chemicals Ltd. (India). Acquisition of PCC was from Guilin Wuhuan Industry Development Co., Ltd.. Al_2_(SO_4_)_3_ used in this study was of analytical grade and used without further purification. Cationic polyacrylamide (CPAM) was supplied by BASF Corporation. Fully bleached pinewood kraft pulp used in handsheet preparation was made in Canada and beaten to 38 to 40°SR using a Valley beater at 2% consistency. After that, the beaten pulp was thickened to approximately 13% consistency for use.

### Preparation of handsheet and measurement of properties

Al_2_(SO_4_)_3_, carrageenan/guar gum or CPAM was added consistently to a concentration of 0.4 wt% water suspension of pulp fibres and fillers. Handsheets with a target grammage of 70 g/m^2^ (PCC content 20%) were formed by a ZQJ2-200 handsheet former. The handsheets were pressed by a ZQYC-200 handsheet press at 4 MPa for 1 min and dried by a DB-3 temperature control electric heating plate at 80℃ for 10 min. All handsheets were conditioned at 23℃ and 50% RH for at least 24 h. The mechanical properties (tensile, bursting) of the handsheet were measured according to TAPPI T494 om-13 and TAPPI T403 om-08, respectively. The filler content was analysed by ashing the paper in a muffler oven according to standard TAPPI method T211.

### Measurement of dynamic drainage and retention

Dynamic drainage tests were performed by a Britt DDJ-2 with a 200-mesh screen and a stirring speed of 750 rpm. 1.54 g fibre and 0.66 g PCC were dispersed in 500 ml of water with a steel agitator. After the furnish, Al_2_(SO_4_)_3_ and carrageenan were added into the drainage jar in sequence and stirred for 30 s. The pinch clamp was loosened and the time was recorded for collecting a volume of 100 ml of the filtrate. The absorbance of the filtrate was measured at a 500 nm wavelength with a Shimadzu UV 2550 spectrometer.

### Instrumental characterization

FT-IR spectra (4000–400 cm^−1^) of handsheets samples were recorded using a Nicolet iS5 spectrometer with iD7 ATR accessory.

SEM observation was performed using a ZEISS EVO 18 scanning electronic microscope. The samples were coated with gold before observation.

#### Statistical analysis method

Three handsheet pieces were prepared for each corresponding variation point. Three readings for tensile index, two readings for bursting index, and one reading for PCC retention were obtained from each handsheet. The n values for tensile index, bursting index and PCC retention were 9, 6 and 3, respectively. Three repeats were made for each variation point in the dynamic drainage tests. The results for each data point or column were expressed as the mean values ±SD.

## Results and discussion

### Single use of carrageenan in PCC filled paper

**Fig 1:**
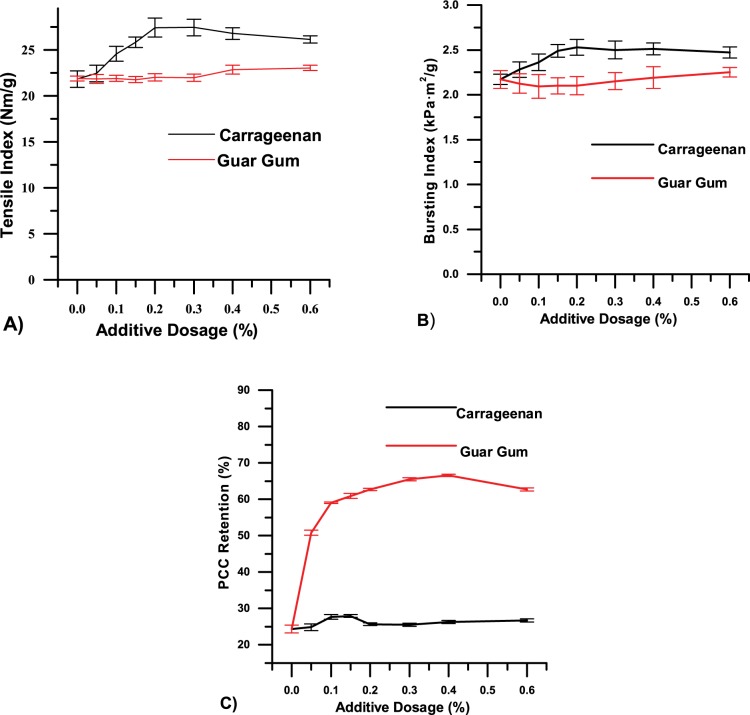
Handsheets of grammage 70 g/m^2^ with 20% PCC as filler at different additive dosages were prepared. A) and B) are, respectively, the tensile and bursting indices as a function of additive dosage; C) is PCC retention as a function of additive dosage.

**Fig 2:**
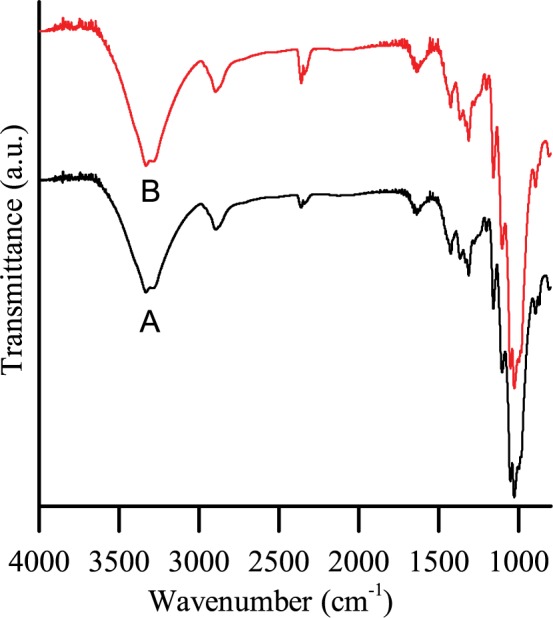
FTIR spectra of Handsheets of grammage 70 g/m^2^ with 20% PCC as filler. A) is spectrum of handsheet with no additive, B) is spectrum of handsheet with carrageenan dosage of 0.2%.

Carrageenan was used alone to assess its effect on paper properties at a PCC addition level of 20%. Gum gum, a polysaccharides composed of D-mannopyranose and D-galactopyranose, was essayed likewise as a contrast because of its structural similarity with carrageenan and mature industrial application in papermaking[13, 14] The results in Fig 1A and 1B show that the tensile and bursting indices of the handsheets increased rapidly with increasing carrageenan dosage. Among the factors contributing to paper strength characteristics, hydrogen bonding was dominant [15]. In the handsheet drying process, the hydroxyl and sulfate groups of carrageenan formed numerous hydrogen bonds with hydroxyl groups of cellulose fibres, especially the sulfate groups of carrageenan, which contributed extra hydrogen bonds compared with hydroxyl [16]. Increment of hydrogen bonding was confirmed by the FTIR detection results showed in Fig 2. After addition of carrageenan, a slight increase in the intensity of the peak at 3200–3600 cm^-1^ was related to the OH vibration, which indicated an increase of hydrogen bonding [17]. Therefore, carrageenan served a function akin to pectins by bridging the gap between cellulose fibres and enhancing the binding force between them. Finally, the strength properties of the handsheet such as tensile and bursting indices were improved. However, due to the finite quantity of cellulose fibres, when the interaction between carrageenan and cellulose fibres reached its limit, additional carrageenan did not change the strength properties [18, 19]. At the optimal carrageenan dosage of 0.2%, the tensile index of the handsheets was 27.41 Nm/g, 25.68% higher than that of handsheets without carrageenan (21.81 Nm/g). The bursting index value was 2.53 kPa·g/m^2^ upon addition of carrageenan, 16.6% higher than handsheets without carrageenan (2.17 kPa·g/m^2^).

With regard to PCC filler retention, results in Fig 1(C) show that with increasing carrageenan dosage, PCC filler retention slightly increased and then decreased to some extent. indicating that carrageenan had little effect on filler retention.

Situation varies for handsheets with guar gum as additive. Commonly, increase of PCC filler retention will lead to decrease of paper mechnical properties; whereas, in the contrast test, PCC retention increased from 25.51% to 62.39% while maintained almost the same tensile/bursting strength for handsheets at the guar gum addition dosage of 0.2%. In contrast to carrageenan, it can be concluded that the guar gum can improve not only paper mechnical strength but also PCC filler retention. The difference is resulted from that guar gum could improve the flocculation state of the cellulose fiber suspension during the sheet-forming process [20], which can’t be achieved by carrageenan due to the anionic sulfate groups on the monosaccharides.

**Fig 3:**
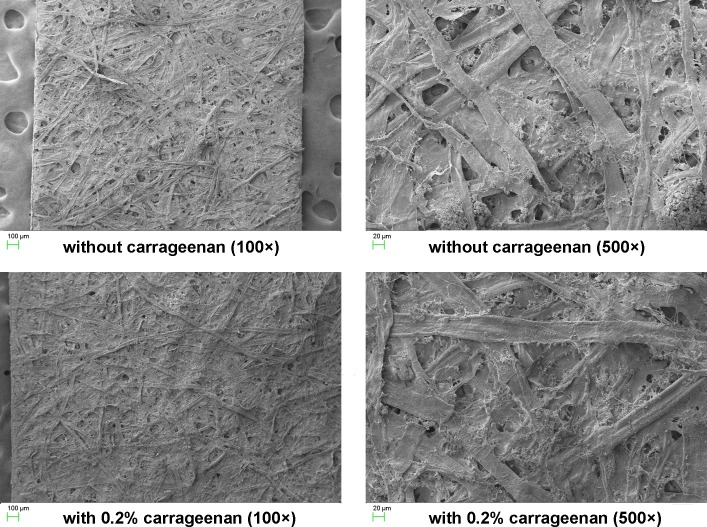
SEM images of handsheets without and with 0.2% carrageenan.

The SEM images in Fig 3 show that the number of pores between fibres appeared to be decreased and the combination between fibres seemed tighter for handsheets with 0.2% carrageenan as additive compared to those without, so the former shaped a smoother and tighter surface. This is likely because carrageenan bridged the gaps between the cellulose fibres [21]. Tighter combination between cellulose fibres normally means higher mechanical strength for paper, which further accounts for the improvements of handsheet mechnical strength.

### Use of carrageenan-CPAM in PCC filled paper

In the papermaking industry, CPAM is widely used as a retention/drainage aid and also in wastewater treatment [22]. As a retention and drainage aid, the commonly used dosage of CPAM is 0.03%-0.08%. Excessive usage of CPAM may cause excessive flocculation of cellulose fibres, and consequently, handsheet formation and drainage deteriorates. The effect of the carrageenan/CPAM binary system was examined at a CPAM dosage of 0.03%.

**Fig 4:**
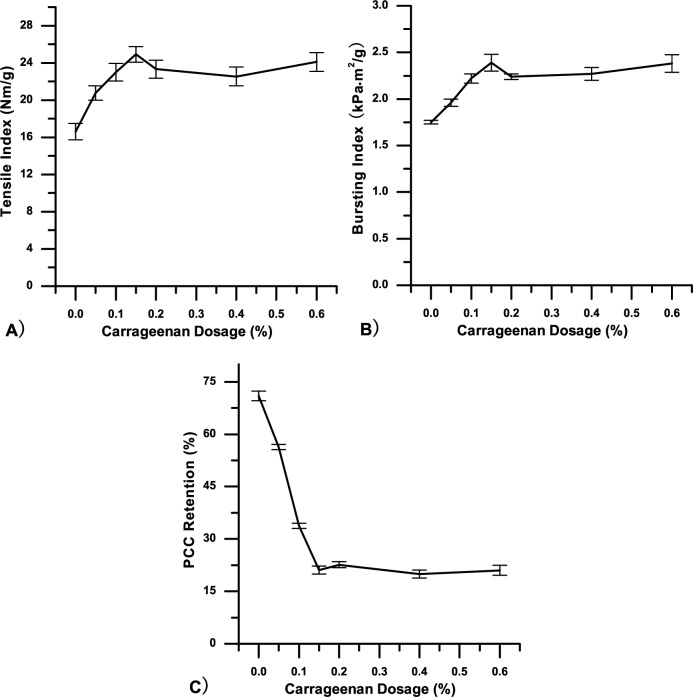
Handsheets of grammage 70 g/m^2^ with 20% PCC as filler, 0.03% CPAM as an additive at different carrageenan dosages were prepared. A) and B) are, respectively, tensile and bursting indices as a function of carrageenan dosage; C) is PCC retention as a function of carrageenan dosage.

Similar to previous single usage observations of carrageenan without CPAM, Fig 4(A) and 4(B) show that with 0.03% CPAM, tensile and bursting indices of handsheets also increased with increasing carrageenan dosage, whereas the tensile index reached a maximum of 24.91 Nm/g at a carrageenan dosage of 0.15%, lower than for handsheets with carrageenan alone. It is assumed that with the CPAM facilitated retention ability, strength enhancement can be achieved at a lower carrageenan dosage.

Fig 4(C) shows that with an increasing carrageenan dosage, PCC filler retention decreased rapidly. At a carrageenan dosage of 0.15%, the PCC retention value was 21.11%, much lower than for handsheets without carrageenan (70.96%). The reason for this phenomenon was that anionic carrageenan neutralized the cationic charges of CPAM, reducing the flocculation of fibres, and thus preventing PCC retention. This result suggests that CPAM can effectively improve retention at low concentrations, while the presence of carrageenan greatly reduced the retention effect of CPAM. Therefore, carrageenan seems not to be suitable for use with the cationic aid CPAM. However, cases are not all identical, as Taggart’s studies showed that subsequent addition of an anionic gum such as algin after the addition of a cationic starch to a pulp slurry could effectively increase retention and paper strength [23]. Accordingly, other DCS (dissolved and colloidal substance) catchers such as Al_2_(SO_4_)_3_, poly AlCl_3_, p-DADMAC or PEO may also work with carrageenan.

### Use of Al_2_(SO_4_)_3_-carrageenan in PCC filled paper

Given the high positive charge of the aluminium ion (+3), chemicals containing aluminium ions such as Al_2_(SO_4_)_3_ are widely used as retention/drainage aids and anionic trash catchers [24]. Hence, Al_2_(SO_4_)_3_ was used with carrageenan to potentially enhance paper strength and improve retention of PCC.

**Fig 5:**
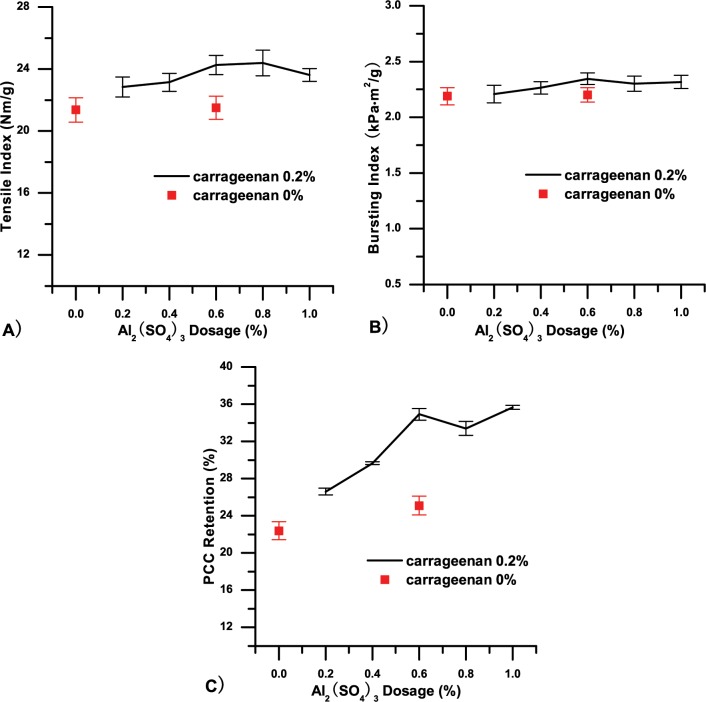
Handsheets of grammage 70 g/m^2^ with 20% PCC as filler, 0.2% & 0% carrageenan as additive at different Al_2_(SO_4_)_3_ dosages were prepared. A) and B) are, respectively, tensile and bursting indices as a function of Al_2_(SO_4_)_3_ dosage; C) is PCC retention as a function of Al_2_(SO_4_)_3_ dosage.

Fig 5(C) shows that at a carrageenan dosage of 0.2%, with an increasing Al_2_(SO_4_)_3_ dosage, PCC filler retention and paper strength increased and reached a plateau at an Al_2_(SO_4_)_3_ dosage of 0.6%. The reasons for this phenomenon were that Al_2_(SO_4_)_3_ was added to the fibres suspension first, and aluminium ions were adsorbed onto the surfaces of the cellulose fibres, producing cationic patches. This led to increased binding sites following the addition of the high molecular weight anionic additive carrageenan. The binding of carrageenan and fibres reduced the gap between fibres and improved filler retention. Normally, increased filler retention results in a reduction of binding between the fibres, and thus, paper strength should decrease; however, Fig 5(A) and 5(B) show that paper strength did not decline. Addition of 0.6% Al_2_(SO_4_)_3_ alone did not cause any apparent change of paper strength, indicating that carrageenan improves the dry strength of handsheets. At an Al_2_(SO_4_)_3_ dosage of 0.6%, the tensile index and PCC retention were 24.25 Nm/g and 34.91%, respectively, representing increases of 13.53% and 55.99% compared to handsheets without additives. It can be inferred from these results that pectins structurally analogous to carrageenan retained by DCS catchers in most papermaking processes likewise contribute to paper strength, although they are regularly troublesome for papermakers.

**Fig 6:**
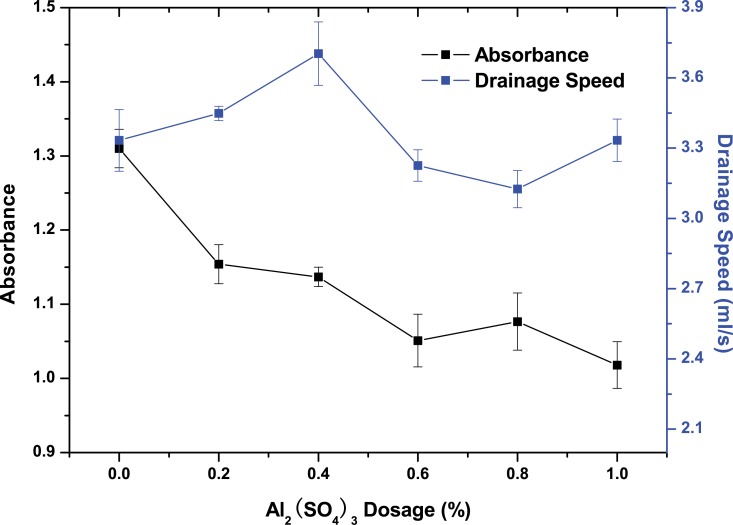
Dynamic drainage tests were performed at a volume of 500 ml of 0.3 w% fibres and 0.13 w% PCC in water. Absorbance of filtrate and drainage speed are functions for Al_2_(SO_4_)_3_ dosage.

Carrageenan dosage in the dynamic drainage tests was optimal at 0.2%. The results in Fig 6 show that with increasing Al_2_(SO_4_)_3_ dosage, filtrate absorbance declined and tended to be unchanged when the Al_2_(SO_4_)_3_ dosage reached 0.6%, which is consistent with the PCC retention curve in Fig 5(C). At an Al_2_(SO_4_)_3_ dosage of 0.6%, the drainage speed was 3.23 ml/s lower than at 0.4%, whereas based on retention results, an Al_2_(SO_4_)_3_ dosage of 0.6% is relatively better for handsheet forming.

### Use of Al_2_(SO_4_)_3_-carrageenan-CPAM in PCC filled paper

**Fig 7:**
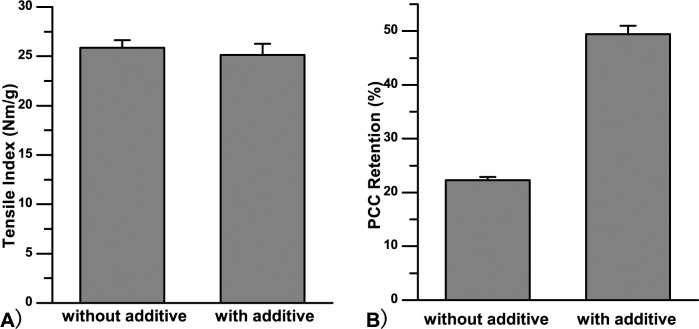
Handsheets of grammage 70 g/m^2^ at a PCC addidtion level of 20%, with Al_2_(SO4)_3_ 0.6%, carrageenan 0.2% and CPAM 0.03% as additives and without additive were prepared. A) and B), respectively, are tensile index and PCC retention of different handsheets.

Based on the results above, adopting the optimum values (dosages of 0.6% Al_2_(SO_4_)_3_, 0.2% carrageenan, and 0.03% CPAM), we prepared handsheets and detected their mechanical strength and PCC filler retention. The results in Fig 7 show that PCC retention increased from 22.33% to 49.45%, while the tensile index was 25.11 Nm/g, almost the same as for handsheets with no additives, indicating that the Al_2_(SO_4_)_3_-Carrageenan-CPAM system seemed to work in strength enhancement and filler retention.

## Conclusions

This study attempted to apply carrageenan in papermaking and extend the range of paper dry strength agents. Applied in papermaking, carrageenan can obviously improve paper strength by strengthening the bonding between cellulose fibres, which was confirmed through FTIR spectra and SEM images. As an anionic dry strength agent, carrageenan decreased the efficiency of some cationic retention aids such as CPAM, thus reducing filler retention. However, carrageenan is suitable for single use or in paper forming processes where Al_2_(SO_4_)_3_ is involved to enhance the strength of paper products and may be potentially used as a new additive in papermaking industry.

## Supporting information

S1 File original data of Fig 1, Fig 4, Fig 5, Fig 6 and Fig 7. (ZIP)
