# Correction: Carrageenan as a dry strength additive for papermaking

**DOI:** 10.1371/journal.pone.0173938

**Published:** 2017-03-08

**Authors:** The PLOS ONE Staff

The affiliation order is incorrect. The affiliations should appear as shown here:

Zhenhua Liu^1,2^, Xinping Li^1^, Wei Xie^2^

**1** College of Bioresources Chemical and Materials Engineering, Shanxi University of Science and Technology, Xi’an, Shanxi Province P. R. China, **2** School of Chemical Engineering, Wuzhou University, Wuzhou, Guangxi Province P. R. China

The publisher apologizes for the error.
